# Author Correction: Air-conditioning and the adaptation cooling deficit in emerging economies

**DOI:** 10.1038/s41467-022-29692-9

**Published:** 2022-04-07

**Authors:** Filippo Pavanello, Enrica De Cian, Marinella Davide, Malcolm Mistry, Talita Cruz, Paula Bezerra, Dattakiran Jagu, Sebastian Renner, Roberto Schaeffer, André F. P. Lucena

**Affiliations:** 1 University of Bologna, Ca’ Foscari University of Venice, Department of Economics, Bologna, Italy; 2 Fondazione CMCC, RFF-CMCC EIEE, Venice, Italy; 3 Ca’ Foscari University of Venice, Department of Economics, Venice, Italy; 4 Harvard University, Ca’ Foscari University of Venice, Department of Economics, Venice, Italy; 5 The London School of Hygiene & Tropical Medicine (LSHTM), Department of Public Health, Environments and Society, London, UK; 6 Centre for Energy and Environmental Economics, Energy Planning Program, Graduate School of Engineering, Universidade Federal do Rio de Janeiro (CENERGIA/PPE/COPPE/UFRJ), Rio de Janeiro, Brazil; 7 Mercator Research Institute on Global Commons and Climate Change, Berlin, German Institute for Global and Area Studies (GIGA), Hamburg, Germany

**Keywords:** Climate-change adaptation, Energy and behaviour

Correction to: *Nature Communications* 10.1038/s41467-021-26592-2, published online 09 November 2021.

The original version of this Article contained an error in Fig. 2, panel a, in which the name of one country, Indonesia, was incorrectly given in place of the correct country name, India. This has been corrected in both the PDF and HTML versions of the Article.

