# Supplementary material for: Air-conditioning and the adaptation cooling deficit in emerging economies
Source: Nat Commun. 2021 Nov 9;12:6460. doi: 10.1038/s41467-021-26592-2 (PMC8578436; doi:10.1038/s41467-021-26592-2)
Supplement: Supplementary file 1 — Supplementary information [file 41467_2021_26592_MOESM1_ESM.pdf]

Air-Conditioning and the Adaptation Cooling Deficit in Emerging Economies

Supplementary Information

Table of Contents

1 Data overview

2 Empirical results

3 Projected changes in air-conditioning and electricity use

1 Data overview

We have built a household-level database using survey data over the period 2002-2018 for four emerging and developing countries - Brazil, India, Indonesia, and Mexico. The aim is to create a homogeneous database, where variables of interest are constructed in a uniform way across four different consumption expenditure surveys. These data have then been combined with sub-national regional climate data.

These four countries have a long tradition in collecting data on household characteristics and behaviors, and conduct expenditure surveys on a regular basis. In Brazil, since 1987, the IBGE (the Brazilian Institute of Geography and Statistics) has conducted household expenditure surveys covering the entire national territory every six or seven years. In India, since 1972-73, the National Sample Survey Office (NSSO) has conducted broad surveys every five years on household expenditures by. The Indonesian Government fields the National Socioeconomic Survey (SUSENAS), a set of large-scale multi-purpose socioeconomic surveys that were initiated in 1963-1964 and have been fielded every year or two since 1993 on a nationally representative level. In Mexico the National Institute of Geography and Statistics (INEGI) has conducted a household survey on income and expenditure every two years since 1992 (less regularly even since 1984).

In every country, surveys run over different years and periods. Supplementary Table 1 summarizes the data availability and the reference period across the four countries considered in this study. The information regarding the period of implementation is important when merging surveys with climate information. As of now, three waves are available for Brazil, Indonesia, and Mexico, including the most recent years (2016-2018), whereas only two waves are available for India.

Two levels of administrative units are included in the database. Level 1 includes administrative units such as States or Union Territories (these are similar to states but managed by the central government). These can be very large, such as Amazonas in Brazil, which has a territorial area of 1,559,000 square kilometers. Level 2 provides the level of administrative division and includes districts or municipalities (Supplementary Table 2). It is available for India, Indonesia, and Mexico. The survey on Brazil does not give information regarding the municipality or district where the households are located, as they want to preserve the identity of the families. States can be further stratified into stratum units: one for rural locations and three for urban locations - the capital of the State, the metropolitan region or other urban municipalities.

The weight variables available in the survey datasets have been used to obtain population-representative descriptive statistics. We have omitted strata and primary sampling unit (PSU) variables, given strong the incompatibilities and missing values among countries and across waves (e.g. in ENIGH 2004 and in all POF waves there are no PSU and strata variables).

The database has been broken down into six main sections describing, respectively, demographics and household characteristics, house features, income and expenditure patterns, energy use, durables ownership, and climate indicators.

For the first two sections, we have selected and harmonized basic information on the households. These include the number of total members and the number of members divided by gender and age. We have considered as adults individuals aged 16 and above, and as infants from newborns to 5 years of age.

To make information on the household heads coherent across surveys, we have elaborated common variables on education level, gender, literacy, and occupation’s type and sector. Education level is based on school attainment data divided among 1) primary, 2) secondary or 3) higher educational levels. We have assumed 0) no education for no or incomplete primary schooling. We have not used data on years of education because none are available for India and Mexico. For the occupation sector we have distinguished between employees in the agricultural sector, including forestry, fishing, livestock, and those not. We have also gathered data on the living area and characteristics of dwellings. These include dummy variables to classify households dwelling in an urban area or not, as well as whether they own the house they live in or not and whether they have electricity access. Common categories across the different countries are identified to homogenize information on energy sources for cooking and lighting, sources of drinking water and types of toilet facilities (Supplementary Table 3).

To compare and summarize information on the characteristics of dwellings across countries and waves,

we have created a set of categorical variables and used them to build a housing index. The housing index is a composite measure of a household's living conditions. It is calculated by using data about assets related to housing construction materials (walls and roof), as well as about the type of toilet facilities and access to drinking water. Each household is assigned a standardized score for each asset, depending on the type and quality of the selected asset. These scores are summed by household, and the final score is then ranked according to three categories, namely Low, Middle and High housing quality. The housing index sets a minimum value of 1 for low quality dwelling conditions, and a maximum value of 3 for high dwelling conditions.

We have gathered data on household income and expenditure. As figures about income are only available for Brazil and Mexico, we have used the total expenditure as a measurement of a household's economic level. Single survey expenditure values are reported in local currency units (LCU) by different reference periods. For each of them, we compute annual values and convert them into constant (2011) purchasing-power-parity (PPP) US dollars. To convert LCU to current USD PPP we have used the World Bank's PPP conversion factor for private consumption[1]. For converting from current USD PPP to 2011 constant USD PPP we have used the CPI Inflation Calculator from the U.S. Bureau of Labor Statistics[2].

Our final dataset includes five expenditure categories (Supplementary Table 4): total expenditure, energy expenditure, electricity expenditure, food expenditure and medical expenditure. Total expenditure is based on aggregate reported values, except for Mexico, for which we noted some inconsistencies affecting the computation of non-monetary expenses across waves (see section on Mexico). To ensure consistency across years, Mexico's total expenditure comprises only its monetary expenditure. We have used total household expenditure in USD2011 PPP to compute total expenditure per adult equivalent (i.e. divided by the number of household members, imposing a weight of 1 for adults and a weight of 0.5 for minors).

Household ownership of a broad number of appliances (including air-conditioning, fans and refrigerators) and other devices (e.g. car, tv, radio) is reported through a set of dummy variables, where 0 = "Not owned" and 1 = "Owned". For each country, we have constructed two variants of climate variables – annual Cooling Degree Days (CDDs), Units: °C days– by using daily dry-bulb and wet-bulb temperature.

We have merged climate and survey data at the district level, since it is the most pinpointed

[1] https://data.worldbank.org/indicator/ PA.NUS.PRVT.PP
[2] https://www.bls.gov/data/inflation_calculator.htm

disaggregated geographical information available for India, Indonesia, and Mexico, except for Brazil, whose data have been merged at the state level (Federal Unit), by stratum type. To do so, first the climate data are aggregated from the grid-cell level to the district (or state) levels utilizing (i) within country administrative boundaries (from geo-spatial shape files) at the respective admin level[3]; and (ii) routines in R (R Core Team, 2018) made available by open-source packages sp, rgdal, raster and spatialEco.

The remainder of this section provides a detailed description of the survey dataset for each country and its climate data.

[3] Admin levels 2 and 3 are for States and Districts, respectively.

Brazil - POF

**Survey name**	Consumer Expenditure Survey (Pesquisa de Orçamentos Familiares - POF)
**Institution**	Brazilian Institute of Geography and Statistics (Instituto Brasileiro de Geografia e Estatística - IBGE)
**Frequency**	Every six or seven years
**Survey waves**	2002-2003, 2008-2009, 2017-2018
**Period of observation**	POF 2002-2003: July 2002 to June 2003. POF 2008-2009: May 2008 to May 2009. POF 2017-2018: June 2017 to July 2018
**Type**	Cross-Sectional
**Coverage**	Occupied permanent private housing units and their residents in the area covered by the survey in both urban and rural areas
**Geographic information**	National, federation units and stratum (1 rural, 3 urban)
**Observation-level**	Household members
**Weighting**	Each household of the subsample is associated with a sample weight or expansion factor that, attributed to the characteristics investigated by the POF, makes it possible to estimate the quantities of interest for the entire population
**Total number of observations**	162,363 (2002-2003: 48,473; 2008-2009: 55,970; 2017-2018: 57,920)
**Data URL**	https://www.ibge.gov.br/estatisticas/sociais/trabalho/9050-pesquisa-de-orcamentos-familiares.html?=&t=downloads

We have concatenated *NÚMERO SEQUENCIAL*[4] (the sequential number for each of the Sample Sectors), *DV DO SEQUENCIAL* (one-digit code that verifies the sequential number assigned to the Sector Sample) and *NÚMERO DO DOMICILIO* (two-digit code, identifying the domicile, assigned sequentially to each household selected in each sector) in order to obtain a unique identifier for each household (*hhid*).

To elaborate descriptive statistics that are population-representative, we have used a weight variable available in the survey. For both waves POF reports two weight variables: *FATOR DE EXPANSÃO 1* and *FATOR DE EXPANSÃO 2*. Both variables identify the expansion factor attributed to the household. The former is the weight used for the survey design; the latter, which is the one we have opt for, is the adjusted weight that should be used to calculate estimates from the survey data.

In the first two waves we have created the dummy variable, *urban*, starting from *ESTRATO GEOGRÁFICO*, to identify whether the household lives in an urban or rural settlement. The variable consists of a sequence of two numbers that identify either the municipality of the capital, the rest of the metropolitan region or the rest of the federative urban units or a rural area. Thus, we associate the first three categories with dwelling in an urban area, while the latter category with dwelling in a rural area. In the 2017-2018 wave we have used the available specific variable *TIPO_SITUAÇÃO_REG*, which takes a value of 1 for urban households and a value of 2 for rural ones.

Aggregate (*total*, *energy*, *electricity* and *food*) expenditures are reported on a monthly basis in local currency (Brazilian real).

In POF waves revenues are reported on monthly basis in local currency. Among the different income variables available in the survey we have opted for *RENDA TOTAL MENSAL DA UC*. This corresponds to the value of the gross monthly income of the consumption unit (family), and it is obtained through the sum of the gross monetary income of all residents of the unit of consumption, obtained through labor, transfers, other income and the positive balance of the financial transaction, plus the portion related to the non-monetary income of the unit of consumption. Then, we compute it on an annual basis in $2011 PPP.

The POF survey considers only employed respondents, without considering those that are inactive or unemployed. POSIÇÃO *NA OCUPAÇÃO* identifies a detailed range of possible positions as principal occupation. We have distributed them among our unique five occupation categories defining the household head’s employment status (see Supplementary Table 3).

[4] Where not otherwise specified we report the original name of the variables common to the greatest number of waves. In some cases, small differences may emerge between waves.

For the other categorical variables we have adopted *SEXO* for a household head’s gender; *CÓDIGO DE ATIVIDADE PRINCIPAL* for the household head’s occupation sector; *CONDIÇÃO DE OCUPAÇÃO* for housing ownership; *MATERIAL QUE PRE- DOMINA NAS PAREDES EXTERNAS* for house walls; *MATERIAL QUE PREDOMINA NA COBERTURA* for house roof; *PROVENIÊNCIA DA ÁGUA (TIPO DE ABASTECIMENTO DE ÁGUA* in 2002) for drinking water; *ESCOADOURO SANITÁRIO* for toilet facilities;

For cooking fuel we have combined the following POF variables:

- *FOGÃO A GÁS*
- *FOGÃO A LENHA*
- *FOGÃO A CARVÃO*
- *FOGÃO A ENERGIA ELÉTRICA*
- *FOGÃO COM OUTRA FONTE*

For electricity access and lighting energy source we have combined the following POF variables:

- *REDE GERAL DE ENERGIA ELÉTRICA*
- *FONTE PRÓPRIA PARA ENERGIA ELÉTRICA*
- *DIESEL/GASOLINA/ GÁS PARA ENERGIA ELÉTRICA*
- *ENERGIA SOLAR PARA ENERGIA ELÉTRICA*
- *ENERGIA EÓLICA PARA ENERGIA ELÉTRICA*
- *ÁGUA PARA ENERGIA ELÉTRICA*
- *BIODIESEL PARA ENERGIA ELÉTRICA*
- *SISTEMA MISTO PARA ENERGIA ELÉTRICA*
- *OUTRA FONTE PARA ENERGIA ELÉTRICA*

In POF 2002 there is no information on house walls, roof materials and toilet type;

India - NSS

**Survey name**	Household Consumer Expenditure, National Sample Survey (NSS)
**Institution**	National Sample Survey Office (NSSO), Ministry of Statistics and Programme Implementation, Government of India
**Frequency**	Quinquennial from 1972-73[5]
**Survey waves**	2004-2005, 2011-2012
**Period of observation**	Over 12 months, from July through June
**Type**	Cross-Sectional
**Coverage**	Randomly selected households based on sampling procedure
**Geographic information**	The whole Indian Union except (i) the Leh (Ladakh) and Kargil districts of Jammu and Kashmir (in 2004-2005), (ii) the interior villages of Nagaland situated beyond five kilometers of the bus route, and villages on the Andaman and Nicobar Islands, inaccessible throughout the year (in 2004-2005 and 2011-2012)
**Observation-Level**	Household members
**Weighting**	The level sample size of State/Union Territories (UT) is allocated between two sectors (urban and rural) in proportion to population as per census 2001
**Total number of observations**	226,306 (2004-2005: 124,644; 2011-2012: 101,662)
**Data URL**	http://microdata.gov.in/nada43/index.php/catalog/central/about

[5] The 2009-2010 survey was repeated in 2011-12 because it was considered a non-normal year.

A unique household identification number (*HHID*) is provided across the different waves. It is a 9 digit sequence, composed by concatenating the First Stage Unit Serial number of the village/urban block (FSU), Hamlet Group Sub Stratum number, Second stage stratum number and Sample household number.

Double weighting is applied to an urban sector subject, provided that urban sample size for larger states not exceed the rural sample size. A variable reporting weights (or multipliers) is given at the end of each record (combined multiplier), and is used to compute population-representative statistics.

Sector is the variable that in the survey identifies households dwelling in a rural or urban area. We use it to create the dummy variable *urban* across all waves.

Information about household members and composition were derivable from demographic variables available in the survey, including *Relation, Sex, Age, HH Size* (respectively *B4 q3, B4 q4, B4 q5, B3 q1* in 2004-2005). We have used them to construct variables on a household head's gender and age, as well as the number of members, adults, children and infants within the household.

No variables reporting income values are available in the survey.

Two reference periods are reported for monthly per capita consumer expenditure (MPCE): a Uniform Reference Period (URP) based on data collected with a 30- day reference period for all items, a Mixed Reference Period (MRP) based on data with a 365-day reference period wherever available, and a 30-day reference period for other items. For the purpose of this work we have used the MRP estimate of per capita consumption (*MPCE MRP*). Expenditure amounts include the sum total of monetary values of all items (i.e. goods and services) consumed by the household on domestic account during the reference period. It is provided on a per capita basis (divided by household size) and in local currency units (Rs).

Monthly household expenditure values are available for energy and electricity. As for food expenditure, values are provided in sub-categories (e.g. cereals, pulses, milk and milk products, etc.) that need to be aggregated. Medical expenditure includes figures on institutional (incurred as an in-patient of a medical institution) and non-institutional expenses.

Categorical variables are constructed starting from the following variables:

- *Education* (*B4 q7* in 2004-2005) for the household head's education level (*edu_head_2);*
- *HH Type code* (*B3 q4* in 2004-2005) for the household head's occupation (*occupation_head*);
- *NIC 2008* (*NIC 1998* in 2004-2005) for the occupation sector of the house- hold head (*sector_head*). NIC codes are provided for each economic activity according to the National

Industrial Classification. We use them to distinguish households working in the agriculture, forestry and fishing sectors (codes starting with 01, 02, 03) from those involved in other economic sectors;

- *Dwelling unit Code* (*B3 q16* in 2004-2005) for house ownership (*ownership_d*); Information on other house characteristics (i.e. size, walls, roofs) is not available,
- *Lighting Code* (*B3 q18* in 2004-2005) to determine whether a household has access to electricity (*ely_access*), and the lighting source (*lighting_source*);
- *Cooking Code* (*B3 q17* in 2004-2005) for the energy sources used for cooking (*cooking_source*);
- Information on sources of drinking water and type of toilet facility are not available;
- Ownership of durables and the respective expenditure are reported for a large number of items (*Whether Possesses and Expenditure Durables* in 2011-2012, *B11 q3, B11 q14* in 2004-2005).

Indonesia - SUSENAS

**Survey name**	National Socio-Economic Survey (Survei Sosial Ekonomi Nasional - SUSENAS)
**Institution**	Indonesian Central Statistics Agency (Badan Pusat Statistik - BPS)
**Frequency**	Irregular since 1963, nationally representative since 1993
**Survey waves**	2004, 2012, 2017
**Period of observation**	July (2004), March to December (2012), March (2017)
**Type**	Cross-Sectional
**Coverage**	Covers a large representative sample of households across Indonesia
**Geographic information**	National, province, regency/city (district), sub-district and village levels. Sub-district and village identification not available after 2012
**Observation level**	Household members
**Weighting**	Frequency weights computed within each stratum (census block which is an administrative/geographical unit) as the inverse of the sampling fraction
**Total number of observations**	648,643 (2004: 65,254; 2012: 286,113; 2017: 297,276)
**Data URL**	https://mikrodata.bps.go.id/mikrodata/index.php/catalog/ SUSENAS

To uniquely identify SUSENAS households across waves (*hhid*), we have used the available variable *urut*. As of 2017, there are 34 provinces (*povinsi*) and 514 districts (*kabupaten/kota*) in Indonesia. However, the number of states and districts has changed in the period 2004-2017. The number of provinces has increased from 30 to 34 and the number of districts from 373 to 514.

To make descriptive statistics that are population-representative, we have used weights available in the survey, namely *wert*;

The variable *b1r5* identifies households dwelling in a rural or urban settlement. We use it to create the dummy variable *urban* across all waves;

In SUSENAS there is no information about income. Total expenditure at monthly level in local currency is reported in the variable *expend;*

There is no total energy expenditure, but single fuel expenditures are available (tri-monthly). They are identified by different codes (*kode*) in variable *b42k6 (electricity = 238, lpg = 242, city gas = 244 kerosene = 246, generator fuel = 248, charcoal= 253, firewood=254);*

For the categorical variables we adopt *jk* for the household head's gender; *b6r3* for housing ownership; *b6r6* for house walls; *b6r5* for house roof; *b6r14a* for electricity access and lighting energy source; *b6r15* for cooking fuel; *b6r9a* for drinking water; *b6r13b* for toilet facilities;

Information on education is available as number of years attended in school. Both education and occupation of the household head are created by using a combination of different variables (*b5r14 to b5r17* for education, *b5r24a1, b5r25, b5r26, b5r31* for occupation);

Information on the ownership of a wide range of goods is provided under variable *b7r4*. Ownership of air conditioning is not available in the 2004 wave, whereas ownership of fans is unavailable in all waves.

Mexico - ENIGH

**Survey Name**	Household Income and Expenditure Survey (Encuesta Nacional de Ingresos y Gastos de los Hogares - ENIGH)
**Institution**	Instituto Nacional de Estadistica, Geografia e Informatica
**Frequency**	Biennial (since 1992)
**Survey waves**	2004, 2012, 2016
**Period of observation**	August to November
**Type**	Cross-Sectional
**Coverage**	It is constituted by households of national or foreign people, who habitually reside in private housing units within the national territory.
**Geographical information**	National, federal entity and municipality levels
**Observation- level**	Household members
**Weighting**	Probability survey weights. Taking account of the weights, the sample is representative at both the national and subnational levels (urban vs rural)
**Total number of observations**	101, 907 (2004: 22, 595; 2012: 9002; 2016: 70, 310)
**Data URL**	https://www.inegi.org.mx/programas/enigh/nc/2016/

There are two versions of the ENIGH survey: the traditional and the new construction. In 2008, INEGI started to publish the results of the survey under a new construction methodology. The main difference among the two versions relies on how revenues are aggregated in reported income variables. For the new construction, the aggregation rules are in accordance with UN recommendations. In our analysis, we have opted for the traditional version for the 2004 wave (since it was the only one available), while we have used the new construction for both the 2012 and the 2016 waves;

There are two identifiers of the respondent, one associated with the house, *folio viv*, and another associated with the household, *folio hog*. We concatenate the two in order to obtain a unique identifier for each household (*hhid*);

In 2004 INEGI collected households and dwelling variables in the same dataset, and these have a unique identifier *FOLIO* - a sequence of 11 digits. The first 10 digits are associated with the dwelling. The last one identifies the households dwelling in that house. If the eleventh digit of *FOLIO* is equal to 0, this refers to the main household; while, if *FOLIO* ends with a digit greater than 0, this refers to other households dwelling in the same house. Since INEGI reports data about dwelling characteristics - e.g. having an air conditioner, house wall materials - only for the main household, we assume that they also apply to the secondary families;

To elaborate descriptive statistics that are population-representative, we have used the weight variable *factor* in the ENIGH survey 2016. In 2012 and in 2004 there were two factors of expansion, *factor viv* and *factor hog* (*HOG* in 2004). Since our analysis is at the household level, we have used *factor hog* as weighting variable;

In all three waves we have created the dummy variable *urban*, starting from *tam loc* (*ESTRATO* in 2004) to identify whether the household dwells in an urban or a rural settlement. This variable identifies urban areas as localities of 2500 or more inhabitants, while rural areas as localities with fewer than 2500 inhabitants;

Aggregate (*total*, *energy*, *electricity* and *food*) expenditures are reported on a tri- monthly basis in local currency (pesos);

As *income* we use the total current income (*ingcor*), which corresponds to the sum of both monetary and non-monetary revenues. Other revenues are reported on a tri-monthly basis in local currency. Thus, we compute annual income and convert it into $2011 PPP through the same procedure as for expenditure;

We construct the *occupation_head* variable through the following procedure:

- Using *id* trabajo (*COD TRAB* in 2004), we only focus on the household-head's primary job occupation;
- We have divided household-heads between those that worked in the previous month and those that did not (*trabajo mp* in 2012-2016; *TRABAJO* in 2004).From inactive respondents, we have removed household-heads who declared to be looking for a job, identifying them as "unemployed" (*act buscot* in 2012-2016; *BUS TRAB* in 2004)Based on their contract, we have distinguished workers between the self-employed, employees and seasonal workers (*indep, subor, tipocontr* in 2012-2016; *POSICION09, CONTR171* in 2004);

For the other category variables we have adopted *sexo jefe* (*SEXO* in 2004) for the household head's gender; *educa jefe* (*ED FORMAL* in 2004) for the household head's education level; *scian* (*SCIAN151* in 2004) for the household head's occupation sector; *tenencia* (*TENENCIA 12* in 2004) for housing ownership; *mat pared* (*MUROS01* in 2004) for house walls; *mat techos* (*TECHOS02* in 2004) for house roof; *disp elect* (*LUZ21* in 2004) for electricity access and lighting energy source; *combustible* (*COMBUS11* in 2004) for cooking fuel; *disp agua* (*AGUA15* in 2004) for drinking water; *sanit agua* (*BANO17* in 2004) for toilet facilities;

There is no information on household energy and electricity consumption in kWh. Hence, we have gathered state-level electricity price information from INEGI's Consumer Price Index[6]. We have then divided households' electricity expenditure by prices to get electricity demand;

For most goods, ENIGH provides the number of durables owned. We use this number to generate the ownership dummy variables.

[6] https://www.inegi.org.mx/programas/inpc/2010/

Climate data

**Data type**	'DegDays 0p25 1970-2018'
**Raw data**	3-hourly temperature (˚C) (GLDAS, Rodell et al., 2004) aggregated to daily timesteps
**Climate variable**	Long-term average Dry (CDD_{db}) and Wet Bulb (CDD_{wb}) Cooling Degree Days
**Frequency**	Annual
**Resolution**	State and District level
**Period of observation**	1970-2016
**Data source**	Mistry M.N., 2019a; Mistry M.N., 2019b

Two climate variables (annual CDD dry-bulb and CDD wet-bulb - *CDDdb* and *CDDwb*) have been computed at the grid-cell level before being spatially aggregated to the district or state/stratum level. Our preferred variable, *CDD wet-bulb,* takes into account the influence of relative humidity on evaporative cooling, which can have an important role in the countries in our study, as they are tropical countries that feature not only spatial heterogeneity in relative humidity, but also coastal regions with high relative humidity. When relative humidity is 100%, dry-bulb and wet-bulb temperature measurements coincide; otherwise wet-bulb temperature (and therefore also CDD *wet-bulb*), are always lower.

Both variants of the CDDs, measured in annual °C days, are assembled by using the input meteorological fields at a high-spatial resolution (0.25° gridded, about 27 km x 27 km at the equator) from the Global Land Data Assimilation System (GLDAS), covering the 1970-2016 period. At each grid-cell the CDDs are calculated by using the American Society of Heating, Refrigerating and Air-Conditioning (ASHRAE) method, and by fixing the baseline temperature at 24 °C, as opposed to the more commonly used threshold of 18°C, which works better for temperate countries. As sensitivity checks, we also use a baseline temperature of 22 °C for both dry- and wet- bulb CDDs.

We have computed long-term climatological averages of degree-days at the administrative level in two ways: 1) starting in 1970 up to the year the survey was conducted, 2) considering the ten years preceding the individual survey years. Since for Brazil and India the survey was carried out over two years, we have opted for the year the wave started - namely, 2002, 2008 and 2017 for Brazil, and 2004, 2011 for India.

We have merged climate and survey data at the district level for India, Indonesia, and Mexico, while for Brazil at the lowest administrative unit at which we can locate households, which is the state (Federal Unit). Within each state, we have been able to distinguish household locations across four strata: rural, capital, metropolitan region, and other urban areas. Gridded CDDs are subsequently aggregated to subnational boundaries in each of the four countries. Subnational population weighted degree-days for the four countries have been assembled by using population data from CIESIN[7]. The 1° gridded populations are matched to the gridded degree-days by using CDO[8] remapping operators, with prior weighting of the degree-days by population to the district level boundaries.

[7] Center for International Earth Science Information Network - CIESIN - Columbia University. 2017. Gridded Population of the World, Version 4 (GPWv4): Population Count, Revision 10. Palisades, NY: NASA Socioeconomic Data and Applications Center (SEDAC). https://doi.org/10.7927/H4PG1PPM.

[8] Schulzweida, Uwe (Max Planck Institute for Meteorologie). 2018. "Climate Data Operators (CDO) User Guide, Version 1.9.0.

Supplementary Table 1: Overview of data availability

	Brazil	India	Indonesia	Mexico
Wave 1	July 2002 - June 2003	July 2004 - June 2005	July 2004	Aug - Nov 2004
Wave 2	May 2008 - May 2009	July 2011 - June 2012	March - Dec 2012	Aug - Nov 2012
Wave 3	June 2017 - July 2018	n.a.	March 2017	Aug - Nov 2016

Supplementary Table 2: Administrative divisions

	Brazil	India	Indonesia	Mexico
Level 1	Federation Units	States & Union Territories	Provinces	Provinces
	27	35/35	30/33/33	32
Level 2	Stratum	Districts	Regencies	Municipalities
	4x27	577/583	360/497/491	490/369/919

Supplementary Table 3: Categorical variables

Variables	Var. Code	Description	Categories
Urban	*urban*	Living or not in an urban area	0 = Rural ; 1 = Urban
Gender Head	*sex_head*	Household head's gender	1 = Male; 2 = Female
Education Head	*edu_head_2*	Household head's education level	0 = No education; 1 = Primary; 2 = Secondary; 3 = Above
Occupation Head	*occupation_head*	Household head's employment status	0 = Inactive; 1 = Unemployed; 2 = Self-employed; 3 = Regular wage earning; 4 = Casual worker; 5 = Other
Sector Head	*sector_head*	Household head's occupation sector	0 = Other sectors; 1 = Agriculture (including forestry, fishing, livestock)
Dwelling Ownership	*ownership_d*	Whether household owns the dwelling where it lives	0 = No; 1 = Yes
House Walls	*house_walls*	Materials of the dwelling's walls	1 = Masonry; 2 = Wood; 3 = Earth structures; 4 = Metal and asbestos; 5 = Waste materials 6 = Other
House Roof	*house_roof*	Materials of the dwelling's roof	1 = Tile; 2 = Concrete; 3 = Wood; 4 = Metal and asbestos; 5 = Earth structure; 6 = Waste materials; 7 = Other
Electricity Access	*ely_access*	Access to electricity	0 = No; 1 = Yes
Lighting Energy Source	*lighting_source*	Energy source for lighting in the dwelling	0 = No lighting; 1 = Public supply/electric utilities; 2 = Renewable plant; 3 = Fossil fuels; 4 = Other
Cooking Energy Source	*cooking_source*	Energy source for cooking	0 = No arrangements; 1 = Electricity; 2 = Gas; 3 = Coal; 4 = Firewood; 5 = Other
Drinking Water Source	*drinking_water*	Source of fresh and drinking water	1 = Piped water; 2 = bottled water; 3 = Wells and springs (including lakes, river); 4 = other
Toilet	*toilet*	Toilet type	0 = No toilet; 1 = Flush; 2 = Pit and latrine; 3 = Other
Housing Index	*housing_index_lab*	Synthesis measure of dwelling conditions	1 = Low quality; 2 = Medium quality; 3 = High quality

Supplementary Table 4: Descriptive statistics. Selected variables in the database

	Brazil 2003		Brazil 2009		Brazil 2018		Mexico 2004		Mexico 2012		Mexico 2016	
	Mean	SD	Mean	SD	Mean	SD	Mean	SD	Mean	SD	Mean	SD
AC	0.01	0.09	0.09	0.28	0.20	0.40	0.04	0.19	0.12	0.33	0.14	0.35
Fan	0.60	0.49	0.62	0.49	0.76	0.43	0.54	0.50	0.47	0.50	0.48	0.50
Refrigerator	0.86	0.35	0.92	0.28	0.98	0.14	0.79	0.41	0.82	0.39	0.86	0.35
Urban	0.85	0.36	0.85	0.35	0.86	0.35	0.77	0.42	0.78	0.41	0.78	0.41
Total exp (USD2011 PPP)	14644.32	22198.35	19344.54	29309.79	22879.01	31150.79	8941.12	10964.58	11309.29	12866.61	12502.30	12813.61
Exp pae (USD2011 PPP)	5550.61	9400.03	7815.43	13358.90	9747.70	14383.69	3031.42	4585.54	4162.75	6021.74	4631.66	5772.92
Energy exp (USD2011 PPP)	506.75	398.59	626.98	555.28	824.58	574.54	468.06	591.98	472.84	597.29	520.85	541.54
Ely exp (USD2011 PPP)	360.08	360.94	465.89	514.86	607.50	528.09	375.11	519.55	274.61	455.34	281.71	422.10
Medical exp (USD2011 PPP)	1243.15	7074.99	1345.80	3168.54	2862.72	4295.45	343.61	1810.12	281.62	1366.05	337.52	1644.36
Food exp (USD2011 PPP)	2745.60	2852.61	3261.98	3234.13	3631.78	3726.63	2730.26	3335.88	3810.63	2789.03	4375.67	3286.14
Electricity consump (kWh)	1766.52	1323.02	1956.01	1551.76	1953.88	1556.37	n/a	n/a	2071.65	2002.14	2098.60	2023.17
N. members	3.63	1.83	3.30	1.65	3.01	1.49	4.04	1.99	3.72	1.88	3.67	1.83
Share under 16	0.24	0.23	0.21	0.23	0.16	0.21	0.28	0.24	0.23	0.23	0.23	0.23
Share infants	0.09	0.15	0.07	0.13	0.06	0.12	0.10	0.15	0.09	0.15	0.08	0.14
Literacy head	0.77	0.42	0.90	0.29	0.85	0.36	0.89	0.31	0.90	0.29	0.92	0.26
House ownership	0.72	0.45	0.73	0.45	0.73	0.45	0.73	0.45	0.68	0.46	0.69	0.46
Electricity access	0.96	0.20	0.98	0.13	0.99	0.07	0.99	0.12	0.99	0.09	1.00	0.07
Housing index	1.48	0.50	2.81	0.43	2.85	0.37	2.81	0.47	2.85	0.42	2.89	0.35
Car	0.30	0.46	0.33	0.47	0.46	0.50	0.27	0.44	0.27	0.44	0.28	0.45
TV	0.89	0.31	0.94	0.23	0.97	0.18	0.92	0.27	0.92	0.27	0.94	0.25
CDDs dry-bulb (24 deg)	529.17	380.66	500.21	391.90	492.15	402.52	343.35	393.56	373.10	414.55	388.31	412.46
CDDs wet-bulb (24 deg)	193.79	203.71	189.03	213.09	187.36	226.64	102.61	158.41	124.80	177.02	118.48	170.27
CDDs dry-bulb (22 deg)	1053.49	583.21	1001.31	598.01	979.50	617.42	593.06	622.46	651.18	652.12	675.89	638.38
CDDs wet-bulb (22 deg)	482.45	339.81	463.06	351.58	452.84	366.18	217.87	292.81	250.09	317.14	240.15	305.29

	Indonesia 2004		Indonesia 2012		Indonesia 2017		India 2005		India 2012	
	Mean	SD	Mean	SD	Mean	SD	Mean	SD	Mean	SD
AC	n/a	n/a	0.05	0.21	0.08	0.27	0.08	0.26	0.12	0.32
Fan	n/a	n/a	n/a	n/a	n/a	n/a	0.52	0.50	0.73	0.45
Refrigerator	0.21	0.41	0.36	0.48	0.54	0.50	0.12	0.33	0.20	0.40
Urban	0.48	0.50	0.50	0.50	0.53	0.50	0.27	0.44	0.32	0.46
Total exp (USD2011 PPP)	3058.30	3820.84	7388.83	9531.56	10058.63	9810.02	3332.24	2810.94	5514.13	5098.54
Exp pae (USD2011 PPP)	969.04	1025.58	2389.18	2843.42	3310.71	3045.11	927.70	822.38	1642.18	1611.93
Energy exp (USD2011 PPP)	312.37	345.24	272.08	247.60	390.86	499.21	323.29	229.89	464.04	307.27
Ely exp (USD2011 PPP)	144.82	180.17	118.79	186.81	248.66	449.01	149.09	183.83	185.62	243.81
Medical exp (USD2011 PPP)	n/a	n/a	210.66	1514.16	425.65	1947.33	308.87	734.28	468.14	1242.36
Food exp (USD2011 PPP)	n/a	n/a	3673.80	2458.50	5120.81	3364.60	1626.25	1025.58	2410.25	1564.59
Electricity consump (kWh)	957.94	1061.26	890.37	8649.57	1392.84	1740.10	798.41	1427.65	931.76	975.85
N. members	3.93	1.69	3.87	1.68	3.76	1.65	4.74	2.41	4.43	2.21
Share under 16	0.27	0.22	0.27	0.21	0.24	0.21	0.31	0.25	0.27	0.24
Share infants	0.10	0.14	0.10	0.14	0.09	0.14	0.11	0.16	0.09	0.14
Literacy head	0.89	0.31	0.92	0.27	0.94	0.23	0.62	0.49	0.68	0.47
House ownership	0.80	0.40	0.80	0.40	0.80	0.40	0.85	0.36	0.84	0.37
Electricity access	0.91	0.29	0.96	0.20	0.98	0.13	0.65	0.48	0.80	0.40
Housing index	2.51	0.57	2.64	0.54	2.79	0.44	n/a	n/a	n/a	n/a
Car	0.05	0.22	0.08	0.27	0.11	0.31	0.02	0.14	0.04	0.20
TV	0.68	0.47	0.09	0.29	0.13	0.34	0.39	0.49	0.59	0.49
CDDs dry-bulb (24 deg)	705.58	472.68	663.42	446.09	658.39	428.99	1037.23	439.16	1045.07	448.12
CDDs wet-bulb (24 deg)	318.59	315.16	327.01	326.62	328.20	327.47	297.15	196.23	299.49	197.71
CDDs dry-bulb (22 deg)	1299.55	630.89	1247.22	607.76	1248.98	585.56	1547.99	567.49	1552.28	577.83
CDDs wet-bulb (22 deg)	678.71	511.54	676.47	515.02	671.57	511.16	527.54	271.48	523.48	271.46

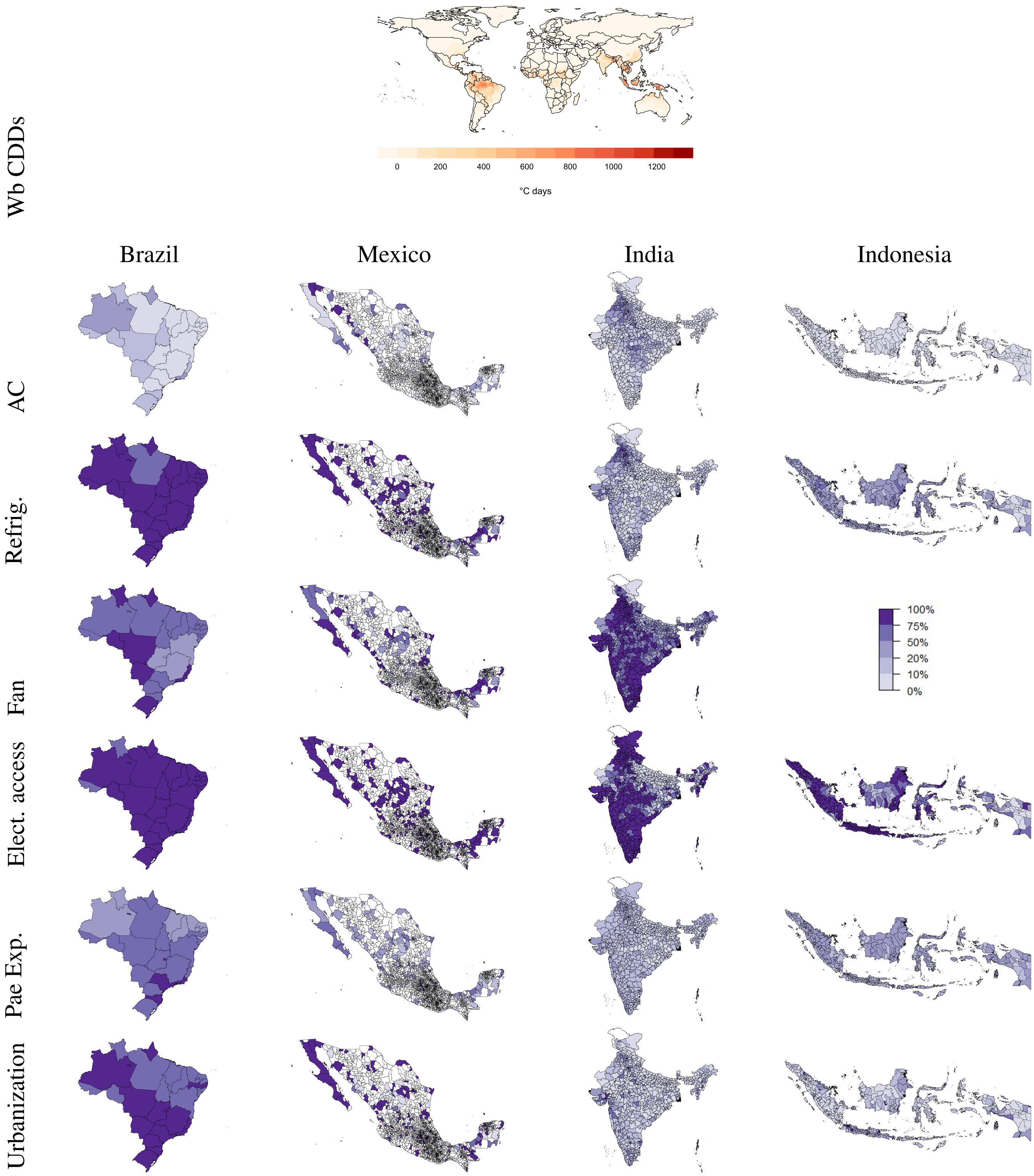

Supplementary Figure 1: Spatial distribution of ownership of AC, fans, refrigerators, per adult equivalent (pae) expenditure, electricity access, urbanization in 2012 (Brazil 2009). Maps are generated using the sp, rgdal, and raster R packages.

2 Empirical results

Supplementary Table 5: Logit models with state fixed effects using CDD wet-bulbs. Marginal Effects

	Brazil			India			Indonesia		Mexico		
	AC	FAN	REF	AC	FAN	REF	AC	REF	AC	FAN	REF
Variables	(1)	(2)	(3)	(4)	(5)	(6)	(7)	(8)	(9)	(10)	(11)
Mean CDD wet-bulb	-0.000277***	0.000455***	0.000111***	-0.000373***	-0.000322*	0.000168	-2.58e-05***	5.20e-05	-0.000609***	0.000929**	9.62e-06
	(0.00006)	(0.00013)	(0.00002)	(8.68e-05)	(0.000173)	(0.000195)	(6.84e-06)	(0.000108)	(0.000223)	(0.000428)	(9.09e-05)
Total Expenditure (Log)	0.0930***	0.102***	0.0212***	0.0558***	0.113***	0.356***	0.0162***	0.423***	0.0277***	0.135***	0.0717***
	(0.00184)	(0.00314)	(0.00072)	(0.00456)	(0.00766)	(0.00946)	(0.000650)	(0.00655)	(0.00341)	(0.00838)	(0.00335)
CDD wb x Log Exp	5.49e-05***	-7.78e-06	-1.31e-05***	5.56e-05***	7.92e-05***	-1.36e-08	4.26e-06***	1.97e-05	8.23e-05***	5.52e-05	7.87e-06
	(0.00001)	(0.00001)	(1.99e-06)	(1.06e-05)	(2.27e-05)	(2.35e-05)	(7.67e-07)	(1.23e-05)	(2.55e-05)	(4.91e-05)	(1.03e-05)
Urban (Yes = 1)				0.0318***	0.0588***	0.129***	0.0145***	0.178***	0.0202***	0.0794***	0.0257***
				(0.00293)	(0.00361)	(0.00485)	(0.000712)	(0.00640)	(0.00467)	(0.0116)	(0.00383)
HH size	-0.0130***	-0.00573***	8.79e-05	-0.00457***	-0.00895***	-0.0284***	-0.00179***	-0.0208***	-0.00695***	-0.0138***	0.00162*
	(0.00079)	(0.00127)	(0.00033)	(0.000404)	(0.000665)	(0.000985)	(0.000101)	(0.00114)	(0.000786)	(0.00219)	(0.000880)
Share < 16	0.0176***	-0.0264***	0.00189	-0.00293	-0.00904*	0.000631	0.00565***	0.166***	0.0182***	-0.0110	0.00255
	(0.00564)	(0.00937)	(0.00212)	(0.00245)	(0.00468)	(0.00721)	(0.000500)	(0.00987)	(0.00377)	(0.0144)	(0.00633)
House Ownership (Yes = 1)	0.0243***	-0.000467	0.00856***	0.0183***	0.0399***	0.0913***	0.00323***	0.127***	0.0186***	0.0328***	0.0403***
	(0.00211)	(0.00364)	(0.00097)	(0.00157)	(0.00465)	(0.00396)	(0.000227)	(0.00559)	(0.00251)	(0.00718)	(0.00342)
Education Head (Primary)	0.0321***	0.0668***	0.00788***	0.0162***	0.0449***	0.0629***	-0.000503**	0.0584***	0.0114***	0.0932***	0.0596***
	(0.00261)	(0.00487)	(0.00127)	(0.00126)	(0.00289)	(0.00250)	(0.000254)	(0.00315)	(0.00131)	(0.00746)	(0.00403)
Education Head (Secondary)	0.0750***	0.0796***	0.0117***	0.0313***	0.0662***	0.151***	0.00572***	0.183***	0.0209***	0.112***	0.0856***
	(0.00309)	(0.00498)	(0.00126)	(0.00205)	(0.00358)	(0.00496)	(0.000354)	(0.00387)	(0.00257)	(0.00837)	(0.00437)
Education Head (Above)	0.133***	0.0228***	0.0117***	0.0527***	0.0669***	0.226***	0.0180***	0.168***	0.0468***	0.148***	0.106***
	(0.00479)	(0.00671)	(0.00175)	(0.00355)	(0.00455)	(0.00569)	(0.000857)	(0.00709)	(0.00481)	(0.0102)	(0.00470)
Age Head	0.000747***	-0.000189	0.000184***	0.000584***	0.000706***	0.00346***	0.000101***	0.00370***	0.000351***	0.00248***	0.00297***
	(0.00007)	(0.00012)	(0.00003)	(5.20e-05)	(9.30e-05)	(0.000149)	(9.09e-06)	(0.000205)	(8.90e-05)	(0.000252)	(0.000113)
Gender Head (Female = 1)	-0.0130***	0.00487	0.00275***	0.0141***	0.0169***	0.0784***	0.000267	-0.00413	-0.00133	-0.00209	0.0148***
	(0.00194)	(0.00328)	(0.00077)	(0.00213)	(0.00251)	(0.00566)	(0.000214)	(0.00383)	(0.00120)	(0.00628)	(0.00220)
House Quality (Medium)	0.0567***	0.285***	0.0377***				0.00267***	0.108***	0.00779***	0.00528	0.0696***
	(0.01128)	(0.03591)	(0.00922)				(0.000604)	(0.00766)	(0.00245)	(0.0284)	(0.0153)
House Quality (High)	0.0661***	0.302***	0.0461***				0.00900***	0.380***	0.0361***	0.0818**	0.133***
	(0.01087)	(0.03557)	(0.00921)				(0.000682)	(0.00887)	(0.00291)	(0.0340)	(0.0162)
Observations	75,290	75,290	75,290	167,648	170,470	166,402	524,112	524,112	78,607	78,607	78,607

Clustered standard errors at district level for MEX, IDN and IND, and robust standard errors for Brazil in parentheses

State fixed effects for India, Indonesia and Brazil. Region fixed effects for Brazil

*** p<0.01, ** p<0.05, * p<0.1

We have also included (but not above-reported) occupation of the household head in MEX and IND regressions

Supplementary Table 6: Logit models with state fixed effects using CDD wet-bulbs. Total Marginal Effects

	Brazil			India			Indonesia		Mexico		
	AC	FAN	REF	AC	FAN	REF	AC	REF	AC	FAN	REF
Variables	(1)	(2)	(3)	(4)	(5)	(6)	(7)	(8)	(9)	(10)	(11)
Mean CDD wet-bulb	0.000245***	0.000381***	-1.27e-05***	9.31e-05***	0.000340***	0.000168***	1.14e-05***	0.000225***	0.000135***	0.00143***	8.05e-05***
	(0.00001)	(0.00002)	(2.41e-06)	(3.19e-05)	(4.22e-05)	(3.67e-05)	(1.30e-06)	(2.30e-05)	(2.37e-05)	(7.35e-05)	(1.70e-05)
Total Expenditure (Log)	0.105***	0.100***	0.0188***	0.0713***	0.134***	0.356***	0.0173***	0.429***	0.0379***	0.142***	0.0726***
	(0.00167)	(0.00245)	(0.00060)	(0.00374)	(0.00438)	(0.00745)	(0.000650)	(0.00465)	(0.00329)	(0.00709)	(0.00299)
Urban (Yes = 1)				0.0320***	0.0586***	0.129***	0.0142***	0.177***	0.0205***	0.0794***	0.0257***
				(0.00294)	(0.00361)	(0.00485)	(0.000700)	(0.00640)	(0.00470)	(0.0116)	(0.00383)
HH size	-0.0131***	-0.00573***	8.91e-05	-0.00459***	-0.00891***	-0.0284***	-0.00175***	-0.0208***	-0.00703***	-0.0138***	0.00161*
	(0.00080)	(0.00127)	(0.00033)	(0.000406)	(0.000663)	(0.000986)	(9.75e-05)	(0.00114)	(0.000797)	(0.00219)	(0.000880)
Share < 16	0.0178***	-0.0264***	0.00192	-0.00295	-0.00900*	0.000631	0.00553***	0.166***	0.0184***	-0.0110	0.00255
	(0.00570)	(0.00938)	(0.00215)	(0.00247)	(0.00467)	(0.00721)	(0.000487)	(0.00987)	(0.00382)	(0.0144)	(0.00632)
House Ownership (Yes = 1)	0.0246***	-0.000467	0.00867***	0.0184***	0.0397***	0.0913***	0.00317***	0.127***	0.0188***	0.0327***	0.0403***
	(0.00214)	(0.00364)	(0.00098)	(0.00158)	(0.00464)	(0.00396)	(0.000222)	(0.00558)	(0.00257)	(0.00718)	(0.00342)
Education Head (Primary)	0.0324***	0.0669***	0.00799***	0.0163***	0.0447***	0.0629***	-0.000493**	0.0584***	0.0115***	0.0932***	0.0596***
	(0.00264)	(0.00488)	(0.00128)	(0.00127)	(0.00289)	(0.00250)	(0.000249)	(0.00315)	(0.00134)	(0.00746)	(0.00404)
Education Head (Secondary)	0.0758***	0.0796***	0.0118***	0.0315***	0.0659***	0.151***	0.00560***	0.183***	0.0212***	0.112***	0.0855***
	(0.00313)	(0.00499)	(0.00127)	(0.00206)	(0.00358)	(0.00495)	(0.000345)	(0.00387)	(0.00263)	(0.00837)	(0.00437)
Education Head (Above)	0.134***	0.0228***	0.0119***	0.0530***	0.0667***	0.226***	0.0176***	0.167***	0.0473***	0.148***	0.105***
	(0.00485)	(0.00671)	(0.00178)	(0.00357)	(0.00455)	(0.00568)	(0.000834)	(0.00709)	(0.00487)	(0.0102)	(0.00471)
Age Head	0.000755***	-0.000189	0.000186***	0.000588***	0.000703***	0.00346***	9.91e-05***	0.00370***	0.000355***	0.00248***	0.00297***
	(0.00007)	(0.00012)	(0.00003)	(5.24e-05)	(9.27e-05)	(0.000149)	(8.85e-06)	(0.000205)	(9.03e-05)	(0.000251)	(0.000113)
Gender Head (Female = 1)	-0.0131***	0.00487	0.00279***	0.0142***	0.0169***	0.0784***	0.000261	-0.00413	-0.00135	-0.00209	0.0148***
	(0.00197)	(0.00328)	(0.00078)	(0.00214)	(0.00251)	(0.00566)	(0.000210)	(0.00383)	(0.00122)	(0.00628)	(0.00220)
House Quality (Medium)	0.0573***	0.285***	0.0381***				0.00262***	0.108***	0.00789***	0.00528	0.0695***
	(0.01142)	(0.03592)	(0.00934)				(0.000591)	(0.00765)	(0.00248)	(0.0284)	(0.0153)
House Quality (High)	0.0669***	0.302***	0.0467***				0.00881***	0.380***	0.0365***	0.0818**	0.133***
	(0.01101)	(0.03557)	(0.00932)				(0.000668)	(0.00887)	(0.00293)	(0.0340)	(0.0162)
Observations	75,290	75,290	75,290	167,648	170,470	166,402	524,112	524,112	78,607	78,607	78,607

Clustered standard errors at district level for MEX, IDN and IND, and robust standard errors for Brazil in parentheses

State fixed effects for India, Indonesia and Brazil. Region fixed effects for Brazil

*** p<0.01, ** p<0.05, * p<0.1

We have also included (but not above-reported) occupation of the household head in MEX and IND regressions

Supplementary Table 7: Standardised logit models with state fixed effects using CDD wet-bulbs. Marginal Effects

	Brazil			India			Indonesia		Mexico		
	AC	FAN	REF	AC	FAN	REF	AC	REF	AC	FAN	REF
Variables	(1)	(2)	(3)	(4)	(5)	(6)	(7)	(8)	(9)	(10)	(11)
Std. Mean CDD wet-bulb	0.0558***	0.0880***	-0.00290***	0.0171***	0.0633***	0.0311***	0.00381***	0.0735***	0.0228***	0.244***	0.0138***
	(0.00152)	(0.00362)	(0.00055)	(0.00585)	(0.00790)	(0.00677)	(0.000434)	(0.00750)	(0.00399)	(0.0126)	(0.00292)
Std. Total Expenditure (Log)	0.0918***	0.0888***	0.0165***	0.0492***	0.0933***	0.247***	0.0126***	0.307***	0.0315***	0.119***	0.0610***
	(0.00145)	(0.00218)	(0.00053)	(0.00258)	(0.00303)	(0.00516)	(0.000475)	(0.00331)	(0.00272)	(0.00596)	(0.00251)
Std. CDD wb x Log Exp	0.0112***	-0.00159	-0.00268***	0.00712***	0.0102***	-1.74e-06	0.000992***	0.00459	0.0118***	0.00793	0.00113
	(0.00121)	(0.00285)	(0.00041)	(0.00136)	(0.00292)	(0.00301)	(0.000179)	(0.00286)	(0.00366)	(0.00706)	(0.00148)
Urban (Yes = 1)				0.0318***	0.0588***	0.129***	0.0145***	0.178***	0.0202***	0.0794***	0.0257***
				(0.00293)	(0.00361)	(0.00485)	(0.000712)	(0.00640)	(0.00467)	(0.0116)	(0.00383)
Std. HH size	-0.0209***	-0.00924***	0.000142	-0.0108***	-0.0212***	-0.0674***	-0.00305***	-0.0354***	-0.0129***	-0.0256***	0.00299*
	(0.00127)	(0.00205)	(0.00053)	(0.000958)	(0.00158)	(0.00233)	(0.000172)	(0.00194)	(0.00145)	(0.00404)	(0.00163)
Share < 16	0.00389***	-0.00583***	0.000419	-0.000674	-0.00208*	0.000145	0.00123***	0.0361***	0.00424***	-0.00256	0.000594
	(0.00125)	(0.00207)	(0.00047)	(0.000564)	(0.00108)	(0.00166)	(0.000109)	(0.00214)	(0.000880)	(0.00336)	(0.00148)
House Ownership (Yes = 1)	0.0243***	-0.000467	0.00856***	0.0183***	0.0399***	0.0913***	0.00323***	0.127***	0.0186***	0.0328***	0.0403***
	(0.00211)	(0.00364)	(0.00097)	(0.00157)	(0.00465)	(0.00396)	(0.000227)	(0.00559)	(0.00251)	(0.00718)	(0.00342)
Education Head (Primary)	0.0321***	0.0668***	0.00788***	0.0162***	0.0449***	0.0629***	-0.000503**	0.0584***	0.0114***	0.0932***	0.0596***
	(0.00261)	(0.00487)	(0.00127)	(0.00126)	(0.00289)	(0.00250)	(0.000254)	(0.00315)	(0.00131)	(0.00746)	(0.00403)
Education Head (Secondary)	0.0750***	0.0796***	0.0117***	0.0313***	0.0662***	0.151***	0.00572***	0.183***	0.0209***	0.112***	0.0856***
	(0.00309)	(0.00498)	(0.00126)	(0.00205)	(0.00358)	(0.00496)	(0.000354)	(0.00387)	(0.00257)	(0.00837)	(0.00437)
Education Head (Above)	0.133***	0.0228***	0.0117***	0.0527***	0.0669***	0.226***	0.0180***	0.168***	0.0468***	0.148***	0.106***
	(0.00479)	(0.00671)	(0.00175)	(0.00355)	(0.00455)	(0.00569)	(0.000857)	(0.00709)	(0.00481)	(0.0102)	(0.00470)
Std. Age Head	0.0119***	-0.00301	0.00292***	0.00793***	0.00958***	0.0469***	0.00137***	0.0502***	0.00562***	0.0397***	0.0476***
	(0.00117)	(0.00193)	(0.00047)	(0.000706)	(0.00126)	(0.00202)	(0.000123)	(0.00278)	(0.00142)	(0.00403)	(0.00181)
Gender Head (Female = 1)	-0.0130***	0.00487	0.00275***	0.0141***	0.0169***	0.0784***	0.000267	-0.00413	-0.00133	-0.00209	0.0148***
	(0.00194)	(0.00328)	(0.00077)	(0.00213)	(0.00251)	(0.00566)	(0.000214)	(0.00383)	(0.00120)	(0.00628)	(0.00220)
House Quality (Medium)	0.0567***	0.285***	0.0377***				0.00267***	0.108***	0.00779***	0.00528	0.0696***
	(0.01128)	(0.03591)	(0.00922)				(0.000604)	(0.00766)	(0.00245)	(0.0284)	(0.0153)
House Quality (High)	0.0661***	0.302***	0.0461***				0.00900***	0.380***	0.0361***	0.0818**	0.133***
	(0.01087)	(0.03557)	(0.00921)				(0.000682)	(0.00887)	(0.00291)	(0.0340)	(0.0162)
Observations	75,290	75,290	75,290	167,648	170,470	166,402	524,112	524,112	78,607	78,607	78,607

Clustered standard errors at district level for MEX, IDN and IND, and robust standard errors for Brazil in parentheses

State fixed effects for India, Indonesia and Brazil. Region fixed effects for Brazil

*** p<0.01, ** p<0.05, * p<0.1

We have also included (but not above-reported) occupation of the household head in MEX and IND regressions

Supplementary Table 8: Logit models with state fixed effects using dry-bulb CDDs. Marginal effects

	Brazil			India			Indonesia		Mexico		
	AC	FAN	REF	AC	FAN	REF	AC	REF	AC	FAN	REF
Variables	(1)	(2)	(3)	(4)	(5)	(6)	(7)	(8)	(9)	(10)	(11)
Mean CDD dry-bulb	-0.000277***	-0.0000410	0.0000345***	-0.000105***	-0.000268***	7.29e-06	-2.61e-05***	-0.000265***	5.97e-07	0.00128***	7.19e-05*
	(0.00003)	(0.00006)	(0.00001)	(3.73e-05)	(5.96e-05)	(8.49e-05)	(5.28e-06)	(7.95e-05)	(2.94e-05)	(0.000140)	(3.97e-05)
Total Expenditure (Log)	0.0794***	0.0858***	0.0206***	0.0396***	0.0790***	0.347***	0.0150***	0.401***	0.0260***	0.168***	0.0731***
	(0.00201)	(0.00337)	(0.00093)	(0.00561)	(0.00938)	(0.0130)	(0.000636)	(0.00807)	(0.00274)	(0.00780)	(0.00311)
CDD db x Log Exp	4.45e-05***	4.07e-05***	-3.46e-06**	2.42e-05***	5.10e-05***	8.98e-06	3.86e-06***	4.61e-05***	1.42e-05***	-8.07e-05***	-2.42e-06
	(3.13e-06)	(0.00001)	(1.39e-06)	(4.29e-06)	(7.79e-06)	(1.02e-05)	(5.85e-07)	(9.12e-06)	(2.87e-06)	(2.01e-05)	(4.30e-06)
Urban (Yes = 1)				0.0310***	0.0548***	0.129***	0.0142***	0.177***	0.0168***	0.0733***	0.0253***
				(0.00243)	(0.00365)	(0.00486)	(0.000670)	(0.00643)	(0.00274)	(0.0110)	(0.00374)
HH size	-0.0123***	-0.00627***	2.72e-06	-0.00449***	-0.00884***	-0.0286***	-0.00182***	-0.0211***	-0.00586***	-0.0143***	0.00167*
	(0.00076)	(0.00126)	(0.00033)	(0.000337)	(0.000637)	(0.000986)	(9.95e-05)	(0.00115)	(0.000620)	(0.00203)	(0.000877)
Share < 16	0.0176***	-0.0210**	0.00220	-0.00229	-0.0105**	8.37e-05	0.00586***	0.168***	0.0147***	-0.00327	0.00253
	(0.00549)	(0.00932)	(0.00217)	(0.00219)	(0.00436)	(0.00716)	(0.000485)	(0.00982)	(0.00279)	(0.0141)	(0.00630)
House Ownership (Yes = 1)	0.0242***	-0.00162	0.00848***	0.0173***	0.0356***	0.0910***	0.00329***	0.128***	0.0142***	0.0308***	0.0401***
	(0.00205)	(0.00361)	(0.00099)	(0.00142)	(0.00444)	(0.00401)	(0.000215)	(0.00563)	(0.00138)	(0.00687)	(0.00337)
Education Head (Primary)	0.0310***	0.0660***	0.00795***	0.0156***	0.0438***	0.0634***	-0.000500**	0.0585***	0.00906***	0.0915***	0.0595***
	(0.00251)	(0.00486)	(0.00128)	(0.00107)	(0.00284)	(0.00253)	(0.000250)	(0.00313)	(0.00110)	(0.00774)	(0.00405)
Education Head (Secondary)	0.0752***	0.0797***	0.0116***	0.0306***	0.0640***	0.151***	0.00566***	0.183***	0.0157***	0.108***	0.0853***
	(0.00301)	(0.00496)	(0.00127)	(0.00192)	(0.00348)	(0.00494)	(0.000340)	(0.00380)	(0.00156)	(0.00874)	(0.00434)
Education Head (Above)	0.131***	0.0253***	0.0115***	0.0516***	0.0656***	0.226***	0.0179***	0.167***	0.0428***	0.143***	0.105***
	(0.00470)	(0.00670)	(0.00178)	(0.00284)	(0.00439)	(0.00567)	(0.000835)	(0.00713)	(0.00380)	(0.0102)	(0.00464)
Age Head	0.000725***	-0.000165	0.000188***	0.000576***	0.000745***	0.00347***	0.000101***	0.00369***	0.000287***	0.00240***	0.00296***
	(0.00007)	(0.00012)	(0.00003)	(4.53e-05)	(8.53e-05)	(0.000149)	(8.86e-06)	(0.000205)	(4.75e-05)	(0.000248)	(0.000111)
Gender Head	-0.0128***	0.00431	0.00274***	0.0146***	0.0178***	0.0789***	0.000222	-0.00464	-0.000300	-0.00144	0.0148***
	(0.00189)	(0.00326)	(0.00078)	(0.00200)	(0.00229)	(0.00561)	(0.000210)	(0.00386)	(0.000973)	(0.00639)	(0.00219)
House Quality (Medium)	0.0539***	0.286***	0.0412***				0.00273***	0.110***	0.00524***	0.0226	0.0694***
	(0.01052)	(0.03704)	(0.00979)				(0.000596)	(0.00751)	(0.00176)	(0.0256)	(0.0153)
House Quality (High)	0.0637***	0.301***	0.0498***				0.00906***	0.381***	0.0259***	0.0934***	0.133***
	(0.01012)	(0.03670)	(0.00978)				(0.000671)	(0.00863)	(0.00332)	(0.0304)	(0.0162)
Observations	75,290	75,290	75,290	167,648	170,470	166,402	525,918	525,918	78,607	78,607	78,607

Clustered standard errors at district level for MEX, IDN and IND, and robust standard errors for Brazil in parentheses

State fixed effects for India, Indonesia and Brazil. Region fixed effects for Brazil

*** p<0.01, ** p<0.05, * p<0.1

We have also included (but not above-reported) occupation of the household head in MEX and IND regressions

Supplementary Table 9: OLS regression models for electricity quantity with state fixed effects using wet-bulb CDDs.

	Brazil	India	Indonesia	Mexico
Variables	(1)	(2)	(3)	(4)
Mean CDD wet-bulb	-0.00220***	-4.24e-05	-0.000549**	-0.00240***
	(0.000181)	(0.000480)	(0.000275)	(0.000326)
Total Expenditure (Log)	0.270***	0.582***	0.490***	0.262***
	(0.00520)	(0.0211)	(0.0107)	(0.00819)
CDD wb x Log Exp	0.000246***	3.26e-05	9.42e-05***	0.000328***
	(1.88e-05)	(5.62e-05)	(2.86e-05)	(3.72e-05)
Urban (Yes = 1)		0.286***	0.266***	0.123***
		(0.0115)	(0.0119)	(0.0140)
HH size	0.0891***	-0.00953***	0.00527***	0.0465***
	(0.00273)	(0.00217)	(0.00189)	(0.00219)
Share < 16	-0.0735***	0.00286	0.126***	-0.0441***
	(0.0182)	(0.0143)	(0.0134)	(0.0152)
House Ownership (Yes = 1)	0.0421***	0.237***	0.119***	0.0710***
	(0.00681)	(0.0103)	(0.00954)	(0.00845)
Education Head (Primary)	0.114***	0.0724***	0.0276***	0.0824***
	(0.0124)	(0.00620)	(0.00627)	(0.00748)
Education Head (Secondary)	0.190***	0.136***	0.104***	0.114***
	(0.0132)	(0.00813)	(0.00733)	(0.00820)
Education Head (Above)	0.261***	0.199***	0.135***	0.160***
	(0.0151)	(0.00938)	(0.0115)	(0.0106)
Age Head	0.00331***	0.00541***	0.00541***	0.00560***
	(0.00023)	(0.000276)	(0.000296)	(0.000261)
Gender Head (Female = 1)	-0.00117	0.0695***	-0.0130**	0.0180***
	(0.00593)	(0.00770)	(0.00546)	(0.00530)
House Quality (Medium)	0.107		0.180***	0.0927***
	(0.127)		(0.0264)	(0.0249)
House Quality (High)	0.0993		0.437***	0.224***
	(0.126)		(0.0301)	(0.0281)
Observations	34,459	85,371	268,310	61,421
R-squared	0.325	0.523	0.395	0.503

Clustered standard errors at district level for MEX, IDN and IND, and robust standard errors for Brazil in parentheses

State fixed effects for India, Indonesia and Brazil. Region fixed effects for Brazil

*** p<0.01, ** p<0.05, * p<0.1

We have also included (but not above-reported) occupation of the household head in MEX and IND regressions

Supplementary Table 10: OLS regression models for electricity quantity with state fixed effects using dry-bulb CDDs.

	Brazil	India	Indonesia	Mexico
Variables	(1)	(2)	(3)	(4)
Mean CDD dry-bulb	-0.00108***	-0.000600***	-0.000459**	-0.00121***
	(8.74e-05)	(0.000198)	(0.000188)	(0.000106)
Total Expenditure (Log)	0.254***	0.498***	0.469***	0.228***
	(0.00584)	(0.0301)	(0.0138)	(0.00682)
CDD wb x Log Exp	0.000127***	8.72e-05***	7.47e-05***	0.000188***
	(9.06e-06)	(2.35e-05)	(2.07e-05)	(1.20e-05)
Urban (Yes = 1)		0.285***	0.263***	0.121***
		(0.0117)	(0.0116)	(0.0114)
HH size	0.0883***	-0.00955***	0.00545***	0.0470***
	(0.00272)	(0.00214)	(0.00186)	(0.00213)
Share < 16	-0.0690***	0.00365	0.128***	-0.0475***
	(0.0182)	(0.0141)	(0.0133)	(0.0147)
House Ownership (Yes = 1)	0.0424***	0.237***	0.120***	0.0670***
	(0.00680)	(0.0102)	(0.00955)	(0.00743)
Education Head (Primary)	0.109***	0.0724***	0.0279***	0.0862***
	(0.0125)	(0.00617)	(0.00614)	(0.00710)
Education Head (Secondary)	0.185***	0.136***	0.103***	0.115***
	(0.0133)	(0.00812)	(0.00724)	(0.00756)
Education Head (Above)	0.258***	0.199***	0.134***	0.167***
	(0.0151)	(0.00945)	(0.0115)	(0.00974)
Age Head	0.00330***	0.00548***	0.00540***	0.00560***
	(0.000228)	(0.000270)	(0.000296)	(0.000257)
Gender Head (Female = 1)	-0.000698	0.0707***	-0.0146***	0.0186***
	(0.00593)	(0.00768)	(0.00539)	(0.00504)
House Quality (Medium)	0.117		0.183***	0.0897***
	(0.125)		(0.0258)	(0.0221)
House Quality (High)	0.106		0.441***	0.218***
	(0.124)		(0.0293)	(0.0244)
Observations	34,459	85,371	269,277	61,421
R-squared	0.326	0.525	0.398	0.525

Clustered standard errors at district level for MEX, IDN and IND, and robust standard errors for Brazil in parentheses

State fixed effects for India, Indonesia and Brazil. Region fixed effects for Brazil

*** p<0.01, ** p<0.05, * p<0.1

We have also included (but not above-reported) occupation of the household head in MEX and IND regressions

Supplementary Table 11: Logit models: sensitivity to omitted variables and waves

	AC-Brazil	AC-Brazil	AC-Brazil	AC-Mexico	AC-Mexico	AC-Mexico	AC-India	AC-India	AC-India	AC-Indonesia	AC-Indonesia	AC-Indonesia
CDD wb	0.000480***	0.000491***	0.000245***	0.000150***	0.000145***	0.000135***	0.000133**	0.000126***	0.0000931***	0.0000303***	0.0000161***	0.0000114***
	(0.00002)	(0.00002)	(0.00001)	(0.00003)	(0.00003)	(0.00002)	(0.00005)	(0.00004)	(0.00003)	(0.00000)	(0.00000)	(0.00000)
Log Tot. exp.	0.220***	0.186***	0.105***	0.0559***	0.0395***	0.0379***	0.134***	0.0995***	0.0713***	0.0485***	0.0253***	0.0173***
	(0.00314)	(0.00364)	(0.00167)	(0.00468)	(0.00348)	(0.00329)	(0.00545)	(0.00496)	(0.00374)	(0.00202)	(0.00094)	(0.00065)
Other vars.	NO	YES	YES	NO	YES	YES	NO	YES	YES	NO	YES	YES
State FE	YES	YES	YES	YES	YES	YES	YES	YES	YES	YES	YES	YES
Year FE	NO	NO	YES	NO	NO	YES	NO	NO	YES	NO	NO	YES
Two waves	NO	NO	YES	NO	NO	YES	NO	NO	YES	NO	NO	YES

Clustered standard errors at district level for MEX, IDN and IND, and robust standard errors for Brazil in parentheses

State fixed effects for India, Indonesia and Brazil. Region fixed effects for Brazil

*** p<0.01, ** p<0.05, * p<0.1

We have also included (but not above-reported) occupation of the household head in MEX and IND regressions.

The specifications that do not use the two waves only use the latest wave.

Supplementary Table 12: Marginal elasticity of air-conditioning to CDDs across temperature measurements and temperature thresholds

	**24 deg - wb**				**24 deg - db**			
	AC-Brazil	AC-Mexico	AC-India	AC-Indonesia	AC-Brazil	AC-Mexico	AC-India	AC-Indonesia
CDDs	0.0565***	0.0230***	0.0172***	0.00373***	0.0608***	0.0533***	0.0444***	0.00339***
	(0.00154)	(0.00406)	(0.00588)	(0.00042)	(0.00194)	(0.00556)	(0.00440)	(0.00034)
Log Tot. exp.	0.0928***	0.0319***	0.0495***	0.0123***	0.0930***	0.0265***	0.0456***	0.0123***
	(0.00148)	(0.00276)	(0.00259)	(0.00046)	(0.00151)	(0.00216)	(0.00247)	(0.00045)
	**22 deg - wb**				**22 deg - db**			
	AC-Brazil	AC-Mexico	AC-India	AC-Indonesia	AC-Brazil	AC-Mexico	AC-India	AC-Indonesia
CDDs	0.0696***	0.0366***	0.0158**	0.00408***	0.0774***	0.0578***	0.0469***	0.00350***
	(0.00177)	(0.00680)	(0.00636)	(0.00040)	(0.00222)	(0.00511)	(0.00469)	(0.00032)
Log Tot. exp.	0.0928***	0.0299***	0.0493***	0.0120***	0.0935***	0.0236***	0.0450***	0.0120***
	(0.00148)	(0.00261)	(0.00262)	(0.00044)	(0.00149)	(0.00238)	(0.00252)	(0.00042)

Clustered standard errors at district level for MEX, IDN and IND, and robust standard errors for Brazil in parentheses

State fixed effects for India, Indonesia and Brazil. Region fixed effects for Brazil

*** p<0.01, ** p<0.05, * p<0.1

We have also included (but not above-reported) occupation of the household head in MEX and IND regressions.

Supplementary Table 13: Total number of households with at least one AC unit around 2040 under different climate change scenarios (in million)

	Historical	RCP4.5	RCP8.5
**Brazil**	10.931	[29.591-36.197]	[35.099-40.377]
**India**	28.904	[142.004-183.668]	[151.822-190.327]
**Indonesia**	5.498	[29.597-40.809]	[31.584-42.564]
**Mexico**	4.795	[9.924-12.300]	[10.536-13.012]

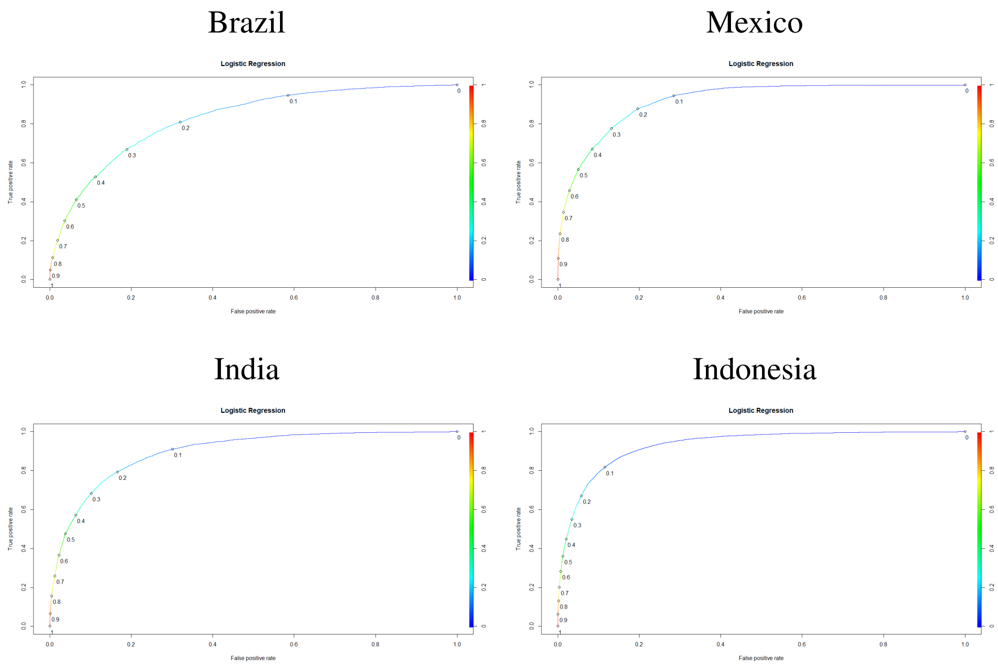

Supplementary Figure 2: Diagnostic of air conditioning logit model performance through the inspection of the Area Under the Receiver Operating Characteristic (ROC) curve.

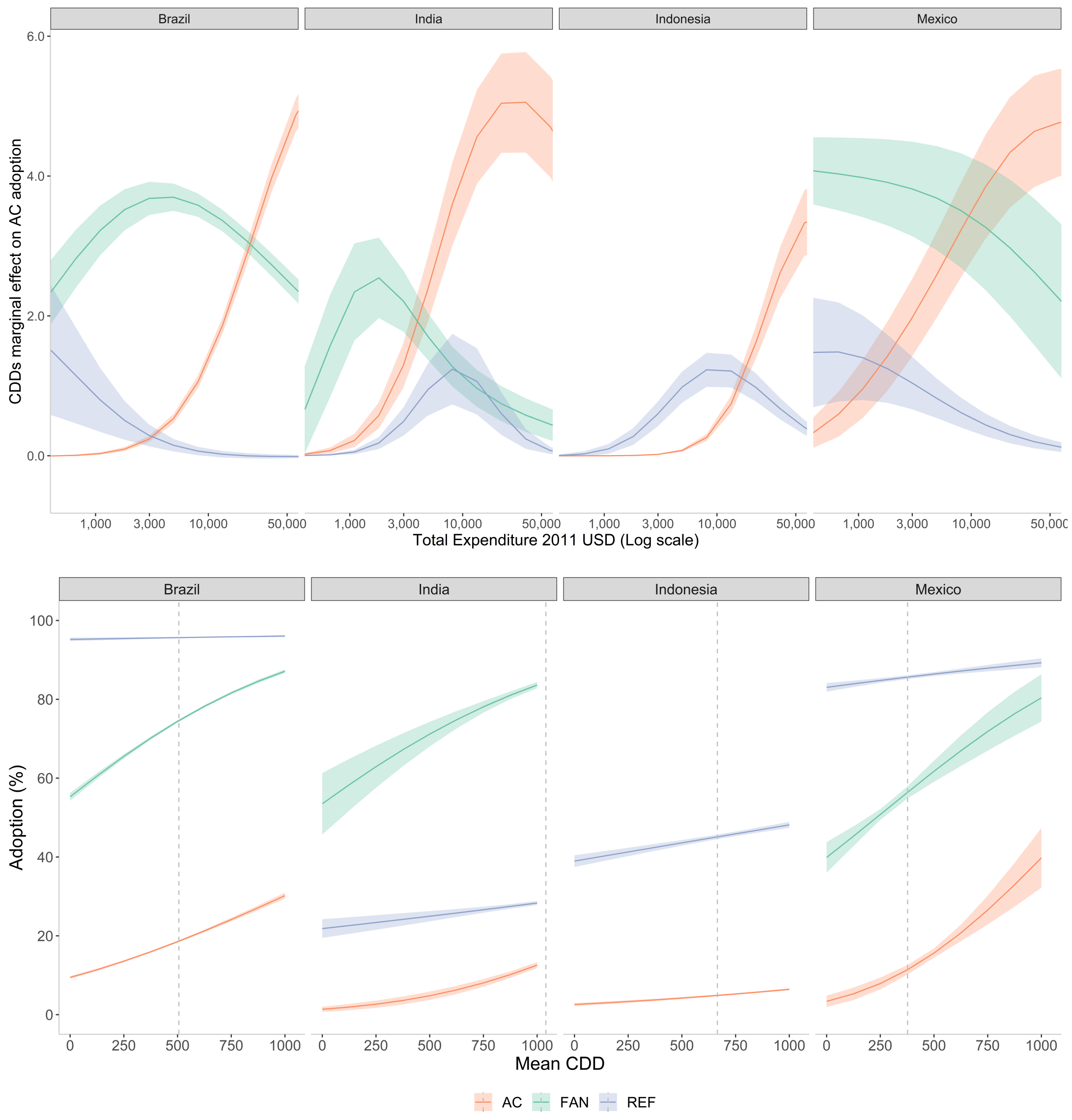

Supplementary Figure 3: Top: Average marginal effect (in percentage points) of dry-bulb CDDs on air conditioner, fan and refrigerator adoption for different levels of total expenditure. Bottom: Predicted adoption rates of air-conditioning and other durable goods used for cooling across different levels of dry-bulb CDDs.

3 Projected changes in air-conditioning adoption and electricity use

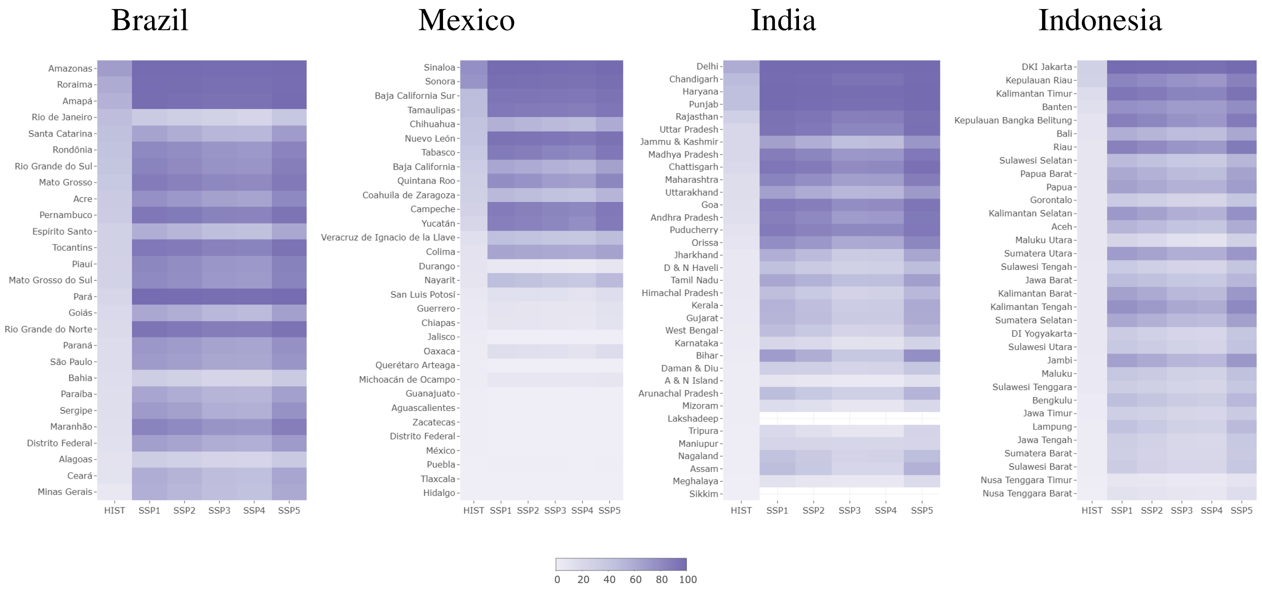

Supplementary Figure 4: Historical and future state-level adoption rates of air conditioners in the year 2040 under RCP4.5

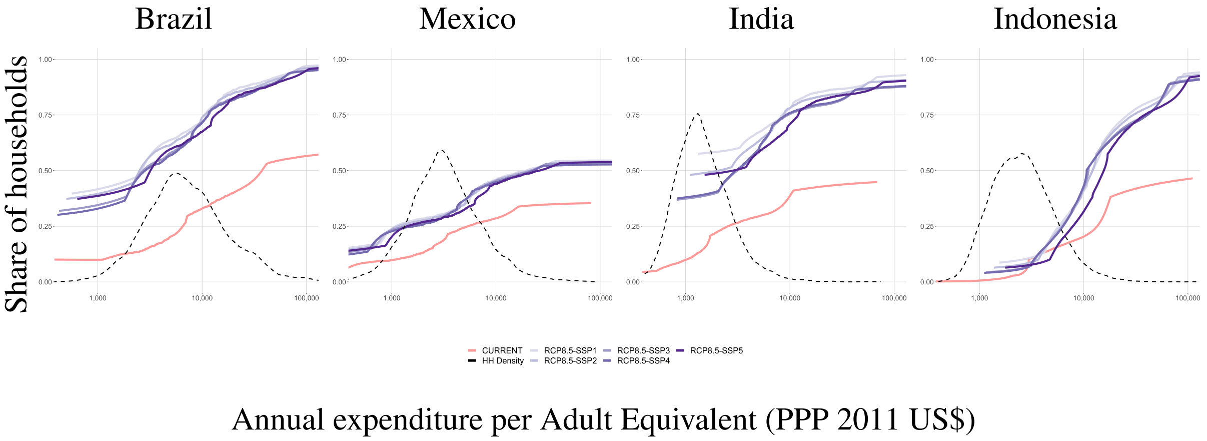

Supplementary Figure 5: Ownership of air conditioners in relation to historical per capita expenditure (2011 US constant dollars at PPP). Adoption scenarios across SSPs

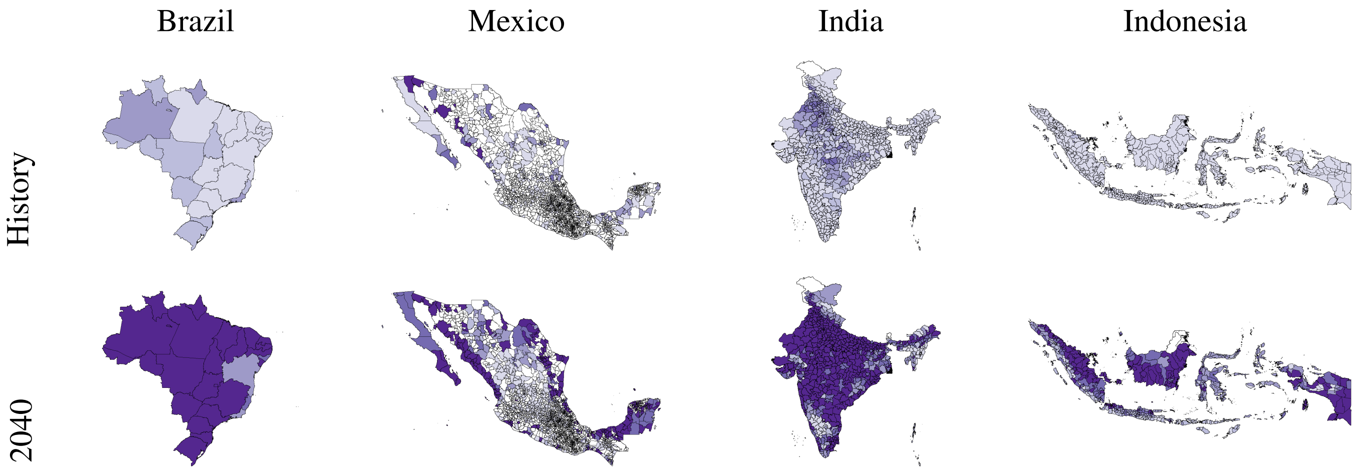

Supplementary Figure 6: Spatial distribution of historical (latest wave) and future (2040, SSP5 RCP 8.5) ownership of AC. Maps are generated using the sp, rgdal, and raster R packages.

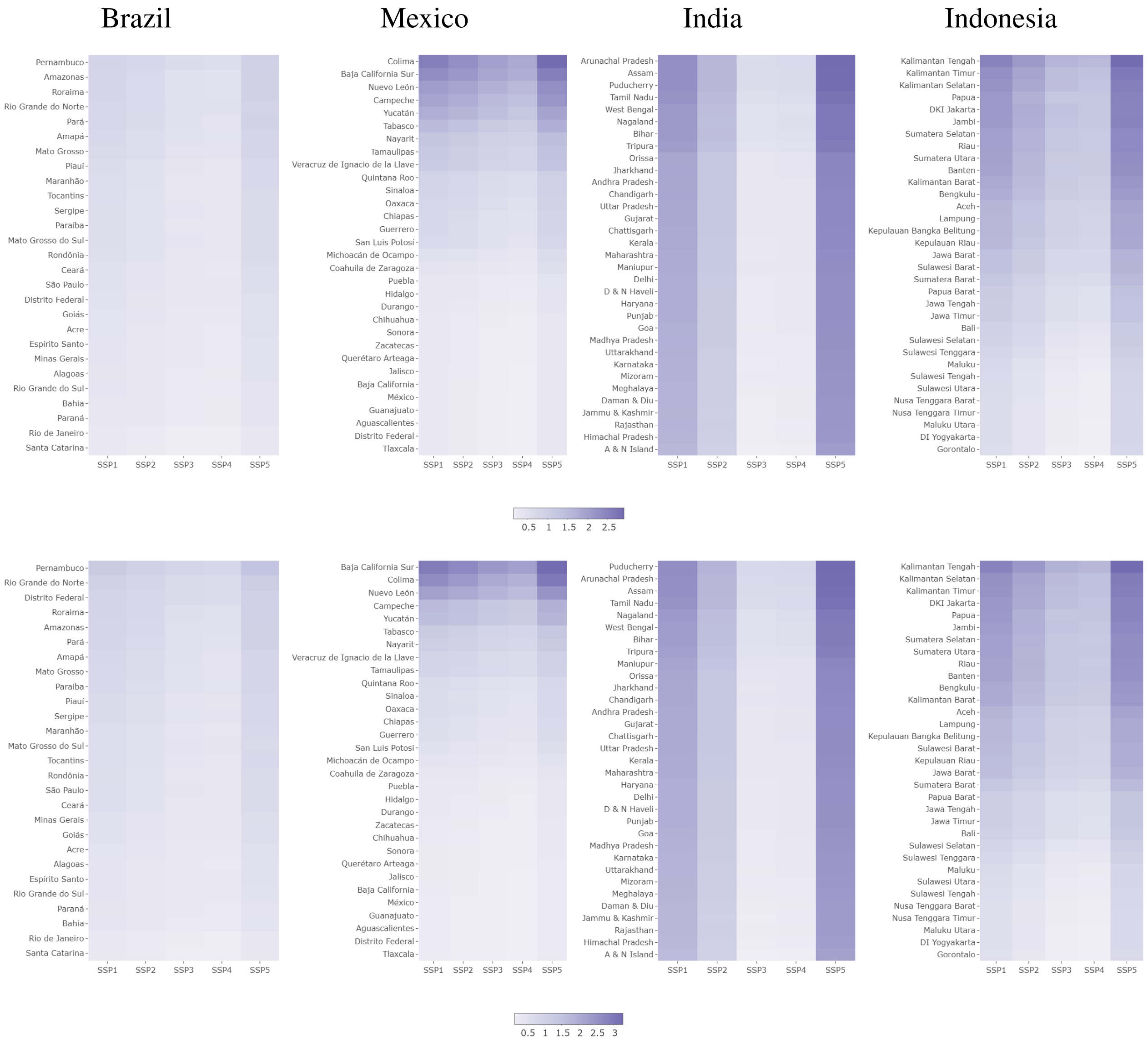

Supplementary Figure 7: Electricity change compared to historical levels - growth factors in the year 2040 under RCP4.5 and RCP8.5

Supplementary Table 14: Growth rate in electricity use: Brazil. Summary statistics computed on state-level averages.

Variable	Obs	Mean	Std. Dev.	Min	Max	P25	P50	P75
RCP85 - SSP1	27	0.686	0.233	0.317	1.298	0.486	0.672	0.819
RCP85 - SSP2	27	0.588	0.202	0.27	1.135	0.417	0.563	0.701
RCP85 - SSP3	27	0.465	0.167	0.209	0.935	0.329	0.441	0.555
RCP85 - SSP4	27	0.437	0.159	0.194	0.894	0.31	0.41	0.522
RCP85 - SSP5	27	0.791	0.267	0.367	1.47	0.566	0.777	0.945
RCP45 - SSP1	27	0.562	0.165	0.292	0.882	0.431	0.57	0.706
RCP45 - SSP2	27	0.48	0.141	0.248	0.77	0.369	0.482	0.603
RCP45 - SSP3	27	0.375	0.112	0.189	0.63	0.289	0.371	0.475
RCP45 - SSP4	27	0.352	0.106	0.176	0.601	0.272	0.345	0.446
RCP45 - SSP5	27	0.65	0.192	0.339	0.999	0.496	0.664	0.815

Note on interpretation. The value of 0.686 represents a 68.6% increase in 2040 compared to the latest wave.

Supplementary Table 15: Growth rate in electricity use: Mexico. Summary statistics computed on state-level averages.

Variable	Obs	Mean	Std. Dev.	Min	Max	P25	P50	P75
RCP85 - SSP1	31	0.775	0.736	0.235	2.934	0.254	0.462	0.989
RCP85 - SSP2	31	0.705	0.679	0.207	2.704	0.229	0.43	0.897
RCP85 - SSP3	31	0.61	0.603	0.169	2.406	0.188	0.368	0.777
RCP85 - SSP4	31	0.557	0.56	0.149	2.245	0.163	0.316	0.713
RCP85 - SSP5	31	0.875	0.821	0.273	3.249	0.293	0.53	1.116
RCP45 - SSP1	31	0.588	0.432	0.235	1.685	0.248	0.395	0.82
RCP45 - SSP2	31	0.534	0.4	0.207	1.531	0.219	0.364	0.745
RCP45 - SSP3	31	0.459	0.357	0.169	1.327	0.18	0.31	0.644
RCP45 - SSP4	31	0.417	0.332	0.149	1.228	0.158	0.267	0.589
RCP45 - SSP5	31	0.666	0.48	0.273	1.904	0.287	0.454	0.926

Note on interpretation. The value of 0.775 represents a 77.5% increase in 2040 compared to the latest wave.

We exclude Morelos from the computation as it is an outlier after the simulation.

Supplementary Table 16: Growth rate in electricity use: India. Summary statistics computed on state-level averages.

Variable	Obs	Mean	Std. Dev.	Min	Max	P25	P50	P75
RCP85 - SSP1	34	2.236	0.133	1.92	2.511	2.146	2.226	2.285
RCP85 - SSP2	34	1.904	0.114	1.631	2.146	1.825	1.896	1.964
RCP85 - SSP3	34	1.427	0.09	1.211	1.618	1.369	1.42	1.489
RCP85 - SSP4	34	1.446	0.091	1.226	1.637	1.385	1.44	1.485
RCP85 - SSP5	34	2.52	0.149	2.169	2.827	2.419	2.508	2.573
RCP45 - SSP1	34	2.172	0.114	1.92	2.41	2.088	2.148	2.201
RCP45 - SSP2	34	1.848	0.097	1.631	2.048	1.779	1.836	1.873
RCP45 - SSP3	34	1.381	0.076	1.211	1.541	1.33	1.372	1.399
RCP45 - SSP4	34	1.4	0.078	1.226	1.566	1.345	1.387	1.418
RCP45 - SSP5	34	2.449	0.127	2.169	2.713	2.354	2.421	2.483

Note on interpretation. The value of 2.236 represents a 223.6% increase in 2040 compared to the latest wave.

Supplementary Table 17: Growth rate in electricity use: Indonesia. Summary statistics computed on state-level averages.

Variable	Obs	Mean	Std. Dev.	Min	Max	P25	P50	P75
RCP85 - SSP1	33	1.966	0.537	1.232	2.931	1.431	2.051	2.459
RCP85 - SSP2	33	1.75	0.476	1.098	2.619	1.277	1.832	2.183
RCP85 - SSP3	33	1.488	0.405	0.931	2.238	1.086	1.562	1.844
RCP85 - SSP4	33	1.438	0.398	0.89	2.148	1.042	1.498	1.796
RCP85 - SSP5	33	2.189	0.599	1.372	3.266	1.592	2.285	2.745
RCP45 - SSP1	33	1.817	0.442	1.228	2.607	1.393	1.808	2.23
RCP45 - SSP2	33	1.617	0.391	1.095	2.329	1.242	1.608	1.961
RCP45 - SSP3	33	1.373	0.332	0.928	1.987	1.055	1.368	1.654
RCP45 - SSP4	33	1.325	0.326	0.887	1.906	1.013	1.321	1.63
RCP45 - SSP5	33	2.024	0.493	1.369	2.906	1.55	2.013	2.486

Note on interpretation. The value of 1.966 represents a 196.6% increase in 2040 compared to the latest wave.

Supplementary Table 18: Air-conditioning adoption: Brazil. Summary statistics computed on state-level averages.

Variable	Obs	Mean	Std. Dev.	Min	Max	P25	P50	P75
RCP85 - SSP1	27	0.825	0.17	0.405	0.996	0.766	0.857	0.96
RCP85 - SSP2	27	0.799	0.182	0.363	0.995	0.721	0.844	0.948
RCP85 - SSP3	27	0.759	0.197	0.311	0.992	0.659	0.799	0.912
RCP85 - SSP4	27	0.75	0.199	0.297	0.992	0.646	0.791	0.9
RCP85 - SSP5	27	0.85	0.16	0.444	0.998	0.792	0.891	0.977
RCP45 - SSP1	27	0.743	0.197	0.33	0.993	0.622	0.769	0.915
RCP45 - SSP2	27	0.711	0.207	0.297	0.992	0.581	0.722	0.896
RCP45 - SSP3	27	0.663	0.218	0.253	0.989	0.515	0.664	0.859
RCP45 - SSP4	27	0.652	0.221	0.245	0.986	0.494	0.649	0.846
RCP45 - SSP5	27	0.774	0.187	0.362	0.995	0.67	0.816	0.944

Note on interpretation. The value of 0.825 represents a 82.5% residential air-conditioning market saturation.

Supplementary Table 19: Air-conditioning adoption: Mexico. Summary statistics computed on state-level averages.

Variable	Obs	Mean	Std. Dev.	Min	Max	P25	P50	P75
RCP85 - SSP1	31	0.406	0.396	0	0.982	0.004	0.191	0.911
RCP85 - SSP2	31	0.396	0.391	0	0.977	0.008	0.177	0.894
RCP85 - SSP3	31	0.382	0.383	0	0.972	0.008	0.166	0.864
RCP85 - SSP4	31	0.374	0.379	0	0.97	0.003	0.146	0.843
RCP85 - SSP5	31	0.417	0.4	0	0.987	0.005	0.207	0.928
RCP45 - SSP1	31	0.386	0.389	0	0.978	0.001	0.149	0.859
RCP45 - SSP2	31	0.376	0.383	0	0.976	0.001	0.142	0.844
RCP45 - SSP3	31	0.361	0.375	0	0.97	0.001	0.129	0.812
RCP45 - SSP4	31	0.352	0.369	0	0.967	0.001	0.118	0.775
RCP45 - SSP5	31	0.399	0.396	0	0.984	0.001	0.167	0.889

Note on interpretation. The value of 0.406 represents a 40.6% residential air-conditioning market saturation.

We exclude Morelos from the computation as it is an outlier after the simulation.

Supplementary Table 20: Air-conditioning adoption: India. Summary statistics computed on state-level averages.

Variable	Obs	Mean	Std. Dev.	Min	Max	P25	P50	P75
RCP85 - SSP1	33	0.656	0.278	0.124	1	0.488	0.682	0.917
RCP85 - SSP2	33	0.605	0.289	0.089	1	0.429	0.596	0.887
RCP85 - SSP3	33	0.523	0.301	0.05	0.993	0.306	0.45	0.835
RCP85 - SSP4	33	0.528	0.3	0.053	0.993	0.316	0.459	0.836
RCP85 - SSP5	33	0.691	0.267	0.162	1	0.544	0.73	0.939
RCP45 - SSP1	33	0.623	0.292	0.095	1	0.451	0.632	0.905
RCP45 - SSP2	33	0.571	0.3	0.07	1	0.37	0.566	0.855
RCP45 - SSP3	33	0.488	0.305	0.042	0.993	0.263	0.397	0.79
RCP45 - SSP4	33	0.494	0.305	0.043	0.993	0.273	0.4	0.801
RCP45 - SSP5	33	0.663	0.28	0.132	1	0.477	0.678	0.919

Note on interpretation. The value of 0.656 represents a 65.6% residential air-conditioning market saturation.

Supplementary Table 21: Air-conditioning adoption: Indonesia. Summary statistics computed on state-level averages.

Variable	Obs	Mean	Std. Dev.	Min	Max	P25	P50	P75
RCP85 - SSP1	33	0.568	0.23	0.106	0.994	0.382	0.535	0.725
RCP85 - SSP2	33	0.526	0.233	0.085	0.989	0.333	0.485	0.692
RCP85 - SSP3	33	0.47	0.231	0.066	0.981	0.278	0.435	0.629
RCP85 - SSP4	33	0.458	0.23	0.062	0.979	0.267	0.424	0.627
RCP85 - SSP5	33	0.607	0.225	0.127	0.997	0.429	0.581	0.775
RCP45 - SSP1	33	0.543	0.226	0.104	0.99	0.36	0.498	0.701
RCP45 - SSP2	33	0.499	0.227	0.084	0.984	0.319	0.458	0.658
RCP45 - SSP3	33	0.442	0.223	0.065	0.973	0.27	0.408	0.588
RCP45 - SSP4	33	0.429	0.222	0.062	0.969	0.256	0.399	0.569
RCP45 - SSP5	33	0.584	0.222	0.126	0.994	0.418	0.542	0.732

Note on interpretation. The value of 0.568 represents a 56.8% residential air-conditioning market saturation.

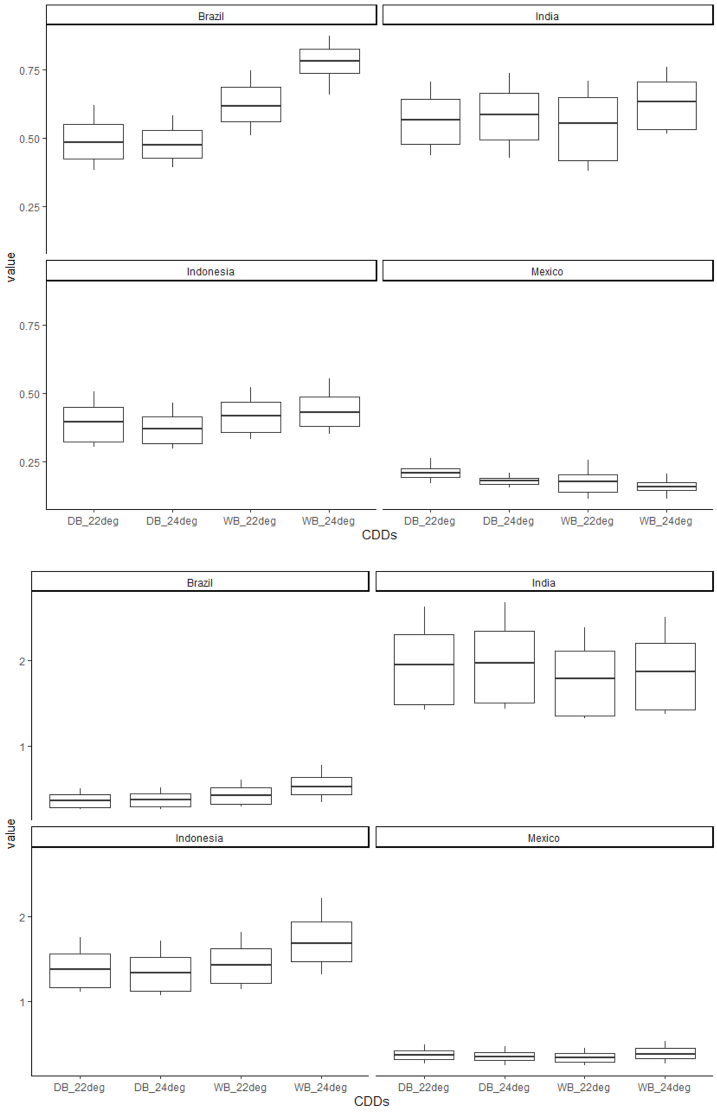

Supplementary Figure 8: Top panel: Predicted air-conditioning adoption rates in 2040 (between 0 and 1, full adoption). Bottom: Predicted growth rates of electricity demand. Sensitivity to temperature thresholds and to temperature measurement.
